# Recombinant Envelope-Proteins with Mutations in the Conserved Fusion Loop Allow Specific Serological Diagnosis of Dengue-Infections

**DOI:** 10.1371/journal.pntd.0004218

**Published:** 2015-11-13

**Authors:** Alexandra Rockstroh, Luisa Barzon, Monia Pacenti, Giorgio Palù, Matthias Niedrig, Sebastian Ulbert

**Affiliations:** 1 Department of Immunology, Fraunhofer Institute for Cell Therapy and Immunology, Leipzig, Germany; 2 Department of Molecular Medicine, University of Padova, Padova, Italy; 3 Robert Koch Institute, Berlin, Germany; University of Texas Medical Branch, UNITED STATES

## Abstract

Dengue virus (DENV) is a mosquito-borne flavivirus and a major international public health concern in many tropical and sub-tropical areas worldwide. DENV is divided into four major serotypes, and infection with one serotype leads to immunity against the same, but not the other serotypes. The specific diagnosis of DENV-infections via antibody-detection is problematic due to the high degree of cross-reactivity displayed by antibodies against related flaviviruses, such as West Nile virus (WNV), Yellow Fever virus (YFV) or Tick-borne encephalitis virus (TBEV). Especially in areas where several flaviviruses co-circulate or in the context of vaccination e.g. against YFV or TBEV, this severely complicates diagnosis and surveillance. Most flavivirus cross-reactive antibodies are produced against the highly conserved fusion loop (FL) domain in the viral envelope (E) protein. We generated insect-cell derived recombinant E-proteins of the four DENV-serotypes which contain point mutations in the FL domain. By using specific mixtures of these mutant antigens, cross-reactivity against heterologous flaviviruses was strongly reduced, enabling sensitive and specific diagnosis of the DENV-infected serum samples in IgG and IgM-measurements. These results have indications for the development of serological DENV-tests with improved specificity.

## Author Summary

The serological diagnosis of dengue is severely complicated by cross-reactivity between antibodies against different flaviviruses. Currently available tests cannot rule out false positive results due to infections or vaccinations with related pathogens such as West Nile virus or Yellow Fever virus. Most cross-reactive antibodies target the conserved fusion loop (FL) domain in the E protein (the major component of the viral envelope). Therefore, we generated insect-cell derived E proteins of DENV from the four different serotypes with mutations in the FL and set up an ELISA-based platform. Using these antigens we were able to detect DENV-infections with high sensitivity. In addition, cross-reactivity with a variety of heterologous flavivirus-infections was eliminated. The results have strong indications for the development of simple and sensitive serological DENV-tests with greatly improved specificity.

## Introduction

Dengue virus (DENV) is a mosquito-transmitted pathogen of the family *Flaviviridae*, a group of small, enveloped and positive stranded RNA-viruses. Besides DENV there are several other human pathogenic vector-borne flaviviruses, such as yellow fever virus (YFV), West Nile virus (WNV), tick-borne encephalitis virus (TBEV) or Japanese encephalitis virus (JEV) [1]. DENV is endemic to over a hundred tropical and subtropical countries worldwide, and the numbers of annual infections are strongly increasing with a current estimate of 400 million [2]. High fever is the most common clinical symptom of a DENV-infection, but also severe complications are observed, such as dengue shock syndrome (DHS) or dengue haemorrhagic fever (DHF). Approx. half a million hospitalizations and several thousands of fatalities are caused by DENV every year [3,4]. Dengue viruses include four major distinct serotypes, named DENV-1 to DENV-4, and survival of an infection with one of these serotypes leads to a lifelong immunity to this serotype, but not to the others [5]. In parallel to the increasing distribution of its vector, mosquitos from the genus *Aedes*, DENV is emerging or re-emerging in several areas including Europe and North America. In Europe local transmission of the virus has been demonstrated in France and Croatia in 2010 [6,7]. In addition, a DENV-outbreak occurred in Madeira in 2012 and resulted in over 2000 cases and transportation of the virus into several other European countries [8].During the acute phase of infection, Dengue can be diagnosed by directly detecting viral RNA or the non-structural protein 1 (NS1), which is secreted by infected cells. About five days after onset of symptoms, these direct infection markers start to decrease in blood and DENV-IgM antibodies appear, followed by IgG a couple of days later. Therefore, except during the acute phase, DENV-infections are usually diagnosed by antibody-measurements, and several serological test systems are available [reviewed in 9,10]. Whereas IgM-detection can indicate recent primary infections, IgG-measurements are useful for detection of secondary infections, where IgM responses usually remain lower, and for serological surveillance activities. Acute dengue infections can be confirmed via a rise in IgM or IgG levels in paired samples [10]. One of the major problems in antibody-based diagnosis of dengue is the similarity of structural proteins of different flaviviruses which leads to cross-reactive antibodies and false positive test results [11–13]. Especially in areas where several flaviviruses co-circulate or in the context of vaccination against e.g. YFV or TBEV, this is of concern [1,14]. As the currently available tests cannot exclude flavivirus cross-reactivity, positive test results have to be confirmed by virus neutralization tests, which are time consuming and require BSL-3 laboratories. The E (envelope) protein is a major target of the human antibody response during DENV infections and is used in most available tests [15]. Cross-reactive antibodies target mainly the highly conserved fusion loop (FL) domain of the E protein which is involved in fusion of the viral and cellular membranes [16–19]. As a consequence, the insertion of mutations into the FL leads to a decrease in binding of cross-reactive antibodies, which has been employed to develop diagnostic methods on the basis of WNV- or JEV- virus-like particles (VLPs) [20–22]. Alternatively, other recombinant antigens than the E protein, such as NS1, have been used to differentiate flavivirus antibodies via titer-determinations [23].

It was shown previously that a bacterially expressed E protein from WNV bearing four point mutations in the FL and an adjacent loop domain can be used to serologically distinguish WNV- from TBEV and DENV-infections [22,24] by reducing the binding of antibodies from heterologous flavivirus infections. Here, we expand this technology to DENV. Quadruple mutant forms of the E proteins from the different DENV-serotypes, containing the same mutations as the WNV-protein [24], were generated in insect cells and analyzed as a diagnostic tool. The results show the high potential of this method for the development of a specific serological DENV-assay.

## Materials and Methods

### Cell culture

*Drosophila S2* cells (Invitrogen) were propagated at 28°C in T-75 cm^2^ flasks in Schneider´s medium supplemented with 10% FCS and 1% Pen/Strep (complete Schneider´s Medium).

### Expression and purification of DENV envelope proteins

The sequences of the DENV-2 E wild type ectodomain (E-protein amino acid residues 1–399; strain 16681) and the quadruple mutants (Equad: T76R, Q77E, W101R; L107R) of DENV serotypes 1–4 (DENV-1: Nauru/West Pac/1974, E-protein amino acid residues 1–399; DENV-3: Sri Lanka/1266/2000, E-protein amino acid residues 1–397; DENV-4: Dominica/814669/1981, E-protein amino acid residues 1–399;) were synthesized (Centic Biotec) and cloned with BglII and EcoRI into the pMT/BiP/V5-His vector (Invitrogen). Plasmids were transfected with a Ca-Phosphate transfection kit (Invitrogen) according to manufacturer´s instructions into *Drosophila S2* cells. To generate stable cell lines 1 μg of pCoHygro (Invitrogen), containing a hygromycin resistance gene, was co-transfected with each expression vector. Stably transfected polyclonal S2 cell populations were generated after 3 weeks of selection with hygromycin B (300 μg/ml) in complete Schneider´s Medium. These cells were then propagated at 28°C in tissue culture flasks with complete Schneider´s medium containing 300 μg/ml hygromycin B and adapted to Sf900II medium containing 600 μg/ml hygromycin B. For an expression culture, cells were seeded at a cell density of 2–3 x10^6^ cells/ml in 600 ml Sf900II medium in 2 l baffled Erlenmeyer shaker flasks at 28°C and 90 rpm and were induced with 700 μM CuSo4 at a cell density of 6 x 10^6^ cells/ml. After 7 days the suspension culture was centrifuged for 15 min and 4000 g at 4°C and culture supernatant was concentrated and diafiltrated against His-binding buffer (20 mM sodium phosphate, 500 mM NaCl, 10 mM Imidazole, pH7,4) using Vivaflow 50R TFF cassettes (Sartorius) according to manufacturers´ instructions. The DENV E proteins were purified by immobilized metal affinity chromatography (IMAC) with 5 ml HisTrap FF crude columns (GeHealthcare) and size exclusion chromatography with a 16/600 HiLoad Superdex 200 pg column (GeHealthcare) using the ÄKTA pure 25 l chromatography system (GeHealthcare). Purified proteins were quantified using a BCA protein assay (Pierce). Conformation of the E-proteins and loss of the FL-domain structure in the Equad mutants were verified by binding studies of monoclonal antibodies recognizing DENV-2 epitopes in the domains DI-DII (antibody DV2-44), DIII (DV2-76, DV2-96, DV2-106) and FL (DV2-29) and WNV-specific FL-antibodies (E18 and E60) [25,26] (S1 Table).

### Serum samples

22 DENV-positive serum samples were obtained from Padova University Hospital (Italy). The confirmed DENV cases were international travelers returning from endemic countries with diagnosis of recent infection. 15 DENV-positive serum samples and one negative control were obtained from Seracare Life Sciences (USA), and 8 DENV-infected serum samples and two negative controls were obtained from ZeptoMetrix Corporation (USA). Three DENV-infected and one WNV-infected samples were from the Robert Koch Institute (Berlin) as part of an external quality assessment study [12]. Serum samples positive for DENV antibodies all fulfilled one of the following criteria: (a) positive in DENV-specific RT-PCR, (b) positive for both DENV IgM and IgG, (c) positive in a DENV-specific virus neutralization test (VNT). Serum samples from confirmed WNV-infections as well as sera from TBEV-infected individuals and negative controls were derived from Italy (University of Padova). The WNV-positive samples (IgG and IgM) were from seroprevalence studies, blood donors or patients with WNV- neuroinvasive disease. WNV-infections were confirmed by VNT. TBEV IgG-positive serum samples were selected from a seroprevalence study in forest rangers with a history of confirmed TBEV infection and no previous exposure to DENV. Serum samples from YFV-vaccinated individuals (IgG positive) participating in a randomized controlled vaccination study were obtained from the Robert Koch Institute (Berlin, Germany). These were confirmed by VNT.

### Ethics statement

Ethical approval for all serum samples from Padova University was obtained from the Padova University Hospital ethics committee. The randomized controlled vaccination study for YFV from the Robert Koch Institute was approved by the Charité medical ethics committee. All persons participating in this research provided informed consent and all samples were analyzed anonymously.

### Antibody measurements

Indicated protein amounts of DENV-2 Ewt and Equad or DENV1-4 Equad mixtures were coated overnight on Nunc polysorb plates (Thermo Scientific) in 100 μl coating buffer (15 mM Na2CO3,35 mMNaHCO3 pH 9.6) at 4°C. The plates were washed three times with 350 μl per well of PBS-0,05% Tween and blocked with 200 μl of 5% non-fat milk powder (blocking solution) for 2 h at room temperature. After a second washing step, human sera were diluted 1:100 in 100 μl blocking solution per well and incubated for 1.5 h at room temperature. Following a third washing step, the HRP-conjugated secondary goat anti human IgG (Fisher Scientific, 1:10000 in 100 μl blocking solution per well) or rabbit anti human μ-chain IgM (Dianova, 1:7500 in 100 μl blocking solution per well) antibody was added for 1 h at room temperature. After a fourth washing step, 100 μl TMB substrate (Biozol) per well were incubated for 30 min at room temperature. The reaction was stopped with 50 μl 1 M H_2_SO_4_ and signals were read out at 450 nm with background reduction at 520 nm in a micro plate reader (Infinite M200, Tecan). All tests were performed in duplicates and in two independent experiments. The Panbio Dengue IgG Indirect ELISA and was used according to the manufacturer´s instructions.

### Statistical analysis

All antibody measurements were performed in duplicates in at least two independent experiments. Graphical and statistical analysis of the data in boxplots was carried out using SigmaPlot.

## Results

**Fig 1:**
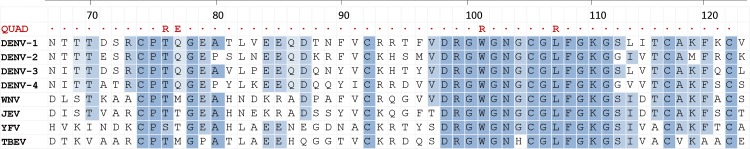
Alignment of the amino acid sequences containing the fusion loop domain from E proteins of DENV 1–4, WNV, JEV, YFV, TBEV and the four point mutations of the QUAD proteins. Amino acids numbering: 1 is start of the E protein.

**Fig 2:**
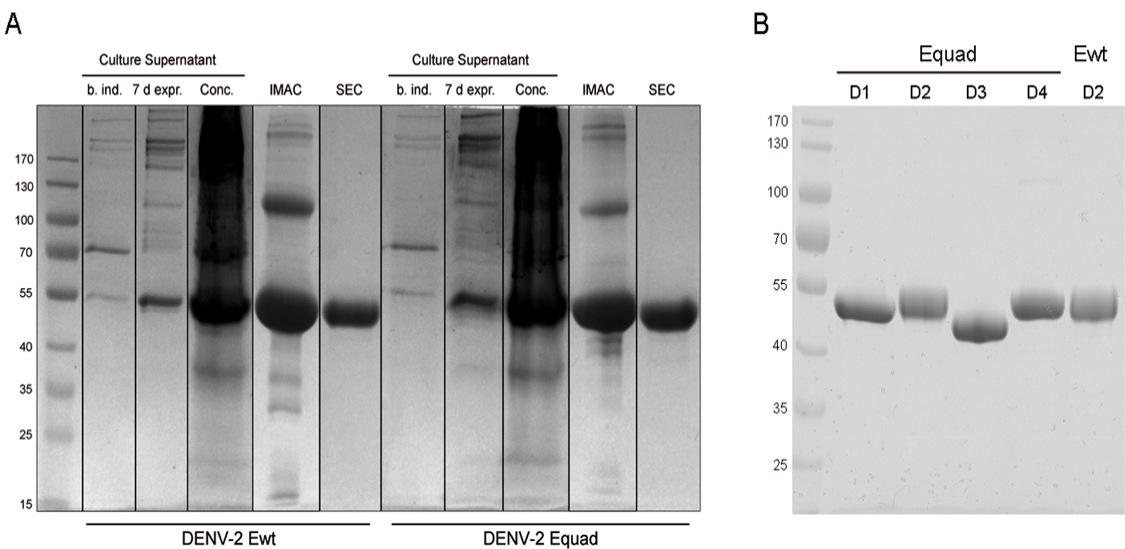
A: Expression and purification of DENV-2 Ewt and Equad from Drosophila S2 culture supernatants; supernatant before induction (b.ind.), after 7 days of expression culture (7 d expr.), concentrated via tangential flow (Conc.) and the two step purification with immobilized imidazole affinity (IMAC) and size exclusion chromatography (SEC) were separated on a 10% SDS-PAGE gel under reducing conditions. B: 6 μg of purified DENV1-4 (D1-D4) Equad and DENV-2 Ewt proteins were analyzed with SDS-PAGE. Proteins were stained with Coomassie blue. Size of molecular weight markers in kilo Daltons is indicated on the left.

To enhance specificity of serological DENV diagnosis we inserted 4 amino acid point mutations into the conserved fusion loop (FL) domain and an adjacent loop domain of DENV wildtype (wt) E proteins, yielding quadruple mutants for DENV serotypes 1 to 4 (DENV 1–4 Equad-proteins, Fig 1). The mutant proteins as well as the DENV-2 wt E-protein were overexpressed in Drosophila S2 cells and secreted into the culture´s supernatant. After purification by immobilized metal affinity chromatography several unspecific proteins were co-purified. Therefore a second purification step using size exclusion chromatography was performed to eliminate the visible unspecific bands (Fig 2A).

**Fig 3:**
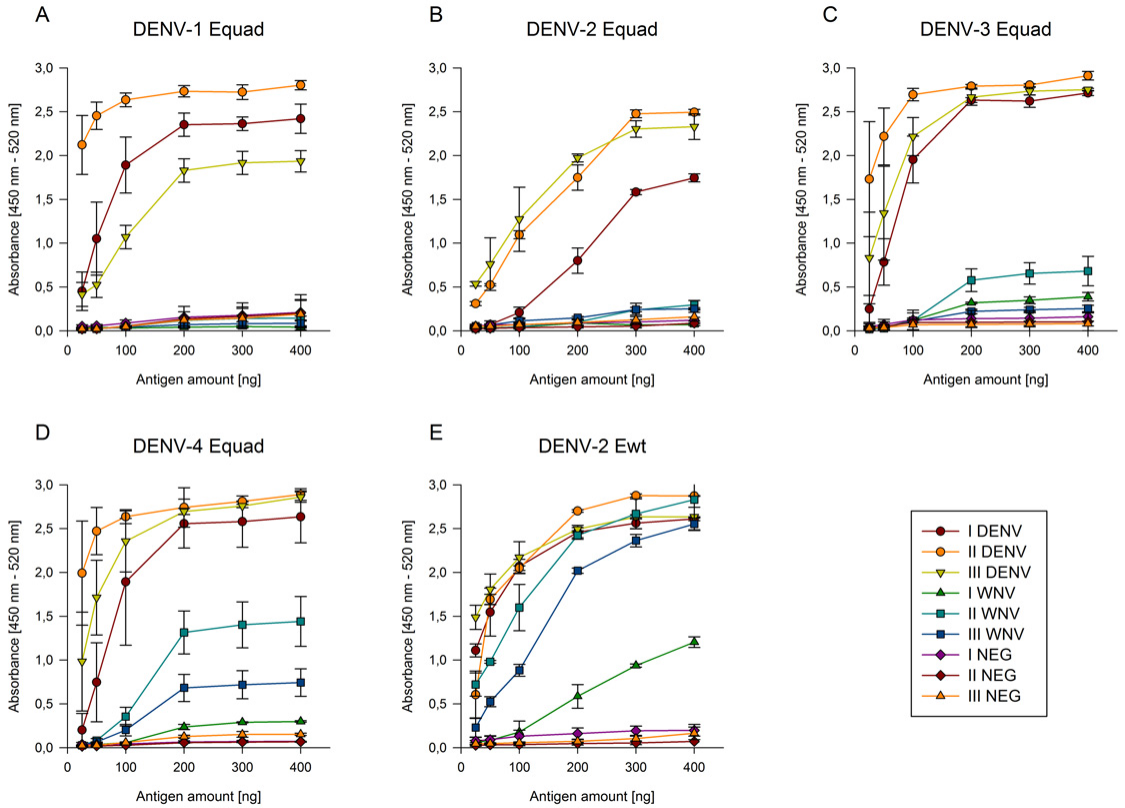
Antigen titration using the indicated amounts of DENV-1 (A), -2 (B), -3 (C) and -4 (D) Equad and DENV-2 Ewt (E) proteins with three different DENV, WNV and negative (NEG) human sera. Measurements were performed in duplicates in at least two independent experiments.

To determine the optimal antigen concentration per well for an IgG-ELISA increasing amounts of the proteins DENV-2 Ewt and DENV 1–4 Equad were incubated with three sera of DENV- and WNV- infected or flavivirus-uninfected individuals, respectively (Fig 3). Signal saturation of DENV-positive sera was observed with an antigen amount of approx. 200 ng per well for DENV -1, 3 and -4 Equad and 300 ng per well for DENV-2 Ewt and Equad proteins. Two of the three DENV-positive sera showed a substantial decrease in binding to the DENV-1 and DENV-2 mutant proteins (Fig 3A and 3B) as compared to the DENV-2 wild type version, but the values were still detectable when 50 ng or more was used. Two WNV-positive samples bound to the wild type antigen as strongly as the DENV-positive sera, and the third started to show such cross-reactivity with more than 200 ng of antigen per well (Fig 3E). However, all WNV sera lost binding almost completely when the mutant antigens were used except for DENV-4 Equad, where two WNV-positive sera still showed substantial signals for 200 ng of antigen per well or more (Fig 3D). Negative sera showed negligible signal intensities for all proteins in all amounts tested. Based on these results an antigen amount of 300 ng per well was chosen for the analysis of a larger serum panel with DENV-2 wt and DENV-2 Equad in an IgG ELISA.

**Fig 4:**
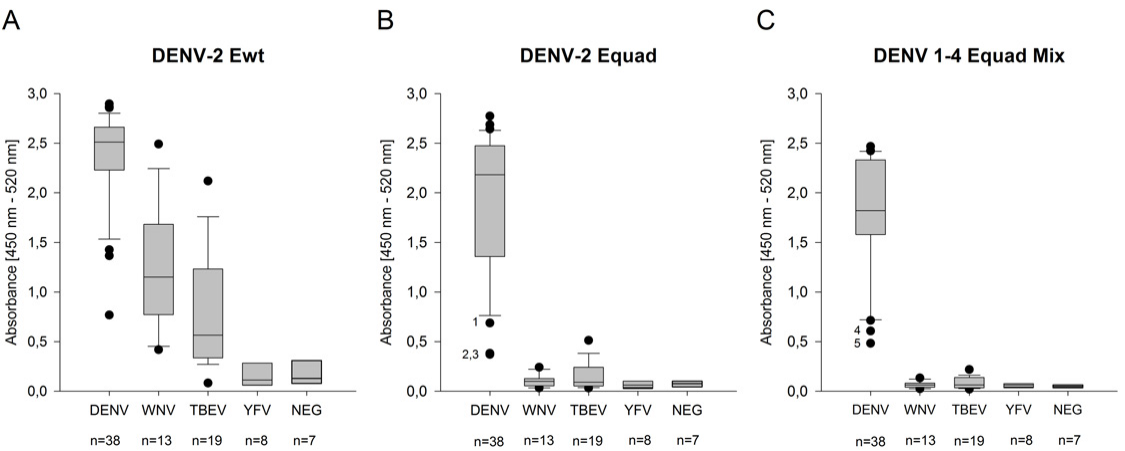
300 ng per well of DENV-2 Ewt (A) and Equad (B) and 160 ng per well of a DENV 1–4 Equad mixture (C) were tested with DENV- WNV- and TBEV- infected and YFV-vaccinated sera compared to negative (NEG) samples in an IgG-ELISA (n = number of individuals). Bottom and top of the boxes are the first and third quartiles. The median signal is depicted as a line inside the box. Whiskers represent the 9^th^ and the 91^st^ percentile. Outliers in B and C are marked with numbers (1–5). Measurements were performed in duplicates in at least two independent experiments.

First, sera from 38 DENV-, 13 WNV-, 19 TBEV- infected, 8 YFV vaccinated and 7 uninfected individuals were incubated with DENV-2 Ewt protein (Fig 4A). Strong binding for DENV-positive samples was observed (mean absorbance value 2.36), however, several WNV- and TBEV- positive sera (mean values 1.26 and 0.82, respectively) showed cross-reactive signals which were in the range of DENV-infected sera. Samples from YFV-vaccinated individuals showed a reduced cross-reactivity in comparison to WNV- and TBEV-infected serum samples. When using the DENV-2 Equad protein, the cross-reactivities of WNV- and TBEV-infected samples were significantly reduced (mean values 0.106 and 0.160, respectively) (Fig 4B). In addition, the signal intensities of the DENV-positive samples changed. The mean value was reduced to 1.9 and the overall signal range increased from 2.1 to 2.4 in comparison to DENV-2 Ewt. Next, a mixture of the Equad proteins of all four serotypes was prepared (DENV 1–4 Equad Mix). Based on the titration curves (Fig 3) a total amount of 160 ng was found to be optimal in specificity and sensitivity, consisting of 50 ng of DENV 1–3 Equad respectively and 10 ng of DENV-4 Equad. The proportional amount of DENV-4 Equad was reduced compared to the other serotypes because it elicited a higher cross-reactivity with heterologous flaviviral sera (Fig 3D). The mixture was tested with the same serum panel. This resulted in an increase of the 25^th^ percentile from 1.36 to 1.6, demonstrating a higher sensitivity compared to using only DENV-2 Equad. At the same time, cross-reactivity of WNV- and TBEV-positive sera was even further reduced (mean values 0.063 and 0.084, respectively) showing a higher specificity of the test in comparison to using DENV-2 Equad only. The stasticial analysis of the data is shown in S2 Table. The five DENV-positive sera detected as outliers on the lower end of the DENV-panel using the mutant antigens in Fig 4 were numbered. Whereas sera 1, 2 (both unknown DENV serotype infections) and 3 (DENV-3 infection) showed decreased binding to the DENV-2 Equad-protein as compared to the wild type, their signals increased when using the DENV 1–4 Equad mix. Also the signals of samples 4 and 5 (DENV-1 and -2 infections, respectively) decreased with the DENV-2 Equad compared to the wild type but were even lower with the mutant mixture (Fig 4C).

**Fig 5:**
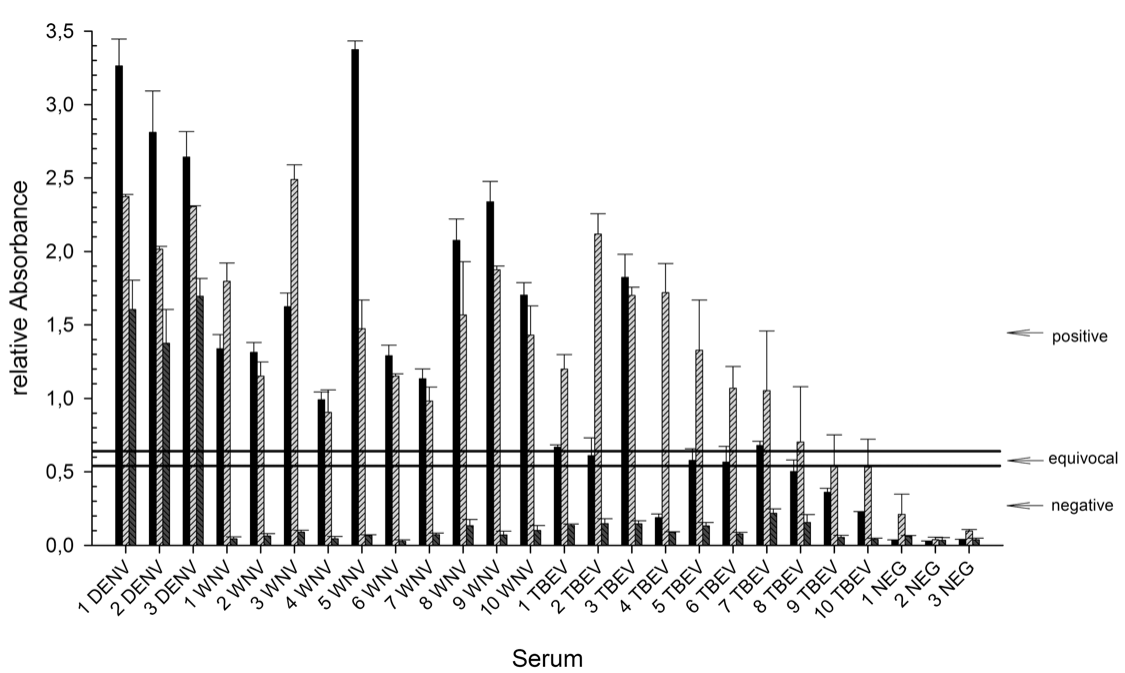
Comparison of different antigens for the detection of DENV IgG. Sera positive for IgG against DENV, WNV, TBEV or negative control sera were analyzed with the Panbio Indirect IgG ELISA (black), the DENV-2 Ewt protein (light gray, lined) or the DENV1-4 Equad mix (dark grey, lined). The absolute absorbance is indicated. Cut-Off values for the Panbio test were obtained by calculation of the internal standard of the manufacturer; these are indicated at the right and only refer to this test (horizontal bars: DENV-positive results with an OD-value higher than 1.1*cut-off, equivocal results having an OD-value between 1.1*cut-off and 0.9*cut-off, negative results with an OD-value lower than 0.9*cut-off).

To compare these results with a state-of-the-art diagnostic method, a number of the serum samples were analyzed with a commercially available DENV-IgG ELISA. The test detected 3/3 DENV-positive sera as positive and 3/3 negative sera as negative. However, 10/10 WNV-infected sera were detected as positive with several OD-values similar to DENV-infected sera. For TBEV-infected sera, 4/10 samples were detected as negative, 3/10 as equivocal and 3/10 as positive (Fig 5). Comparison with the values obtained using DENV Ewt showed a similar cross-reactivity. In contrast, when using the DENV 1–4 Equad mixture high signals were obtained only with the three DENV-infected sera (mean values of 1.5, 1.6 and 1.7, respectively) and cross-reactivity was strongly reduced in all samples from WNV- and TBEV-infections (mean values all >0,3, Fig 5).

**Fig 6:**
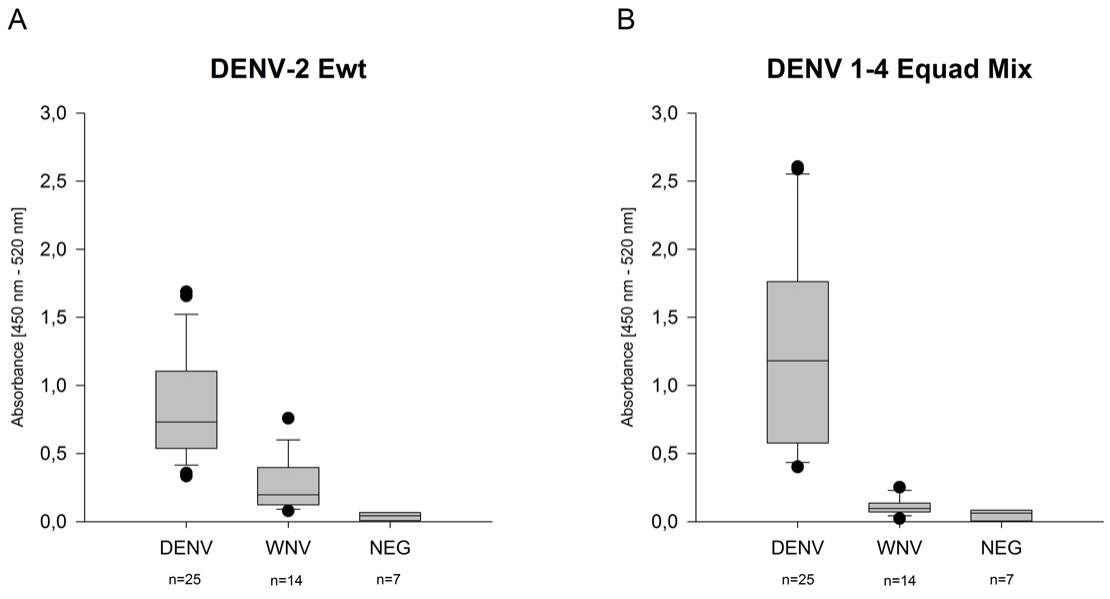
IgM-ELISA on 300 ng of DENV-2 Ewt (A) and DENV 1–4 Equad mixture (B) with different DENV, WNV and negative (NEG) sera (n = number of individuals). Bottom and top of the boxes are the first and third quartiles. The median signal is depicted as a line inside the box. Whiskers represent the 9^th^ and the 91^st^ percentile. Measurements were performed in duplicates in at least two independent experiments.

Subsequently, the proteins were used to measure IgM antibodies in human sera. 300 ng of DENV 1–4 Equad mix were found to be optimal for IgM detection (S3 Table), which was then compared to 300 ng of DENV-2 Ewt in binding of IgM-positive DENV and WNV sera. Generally, the cross-reactivity of heterologous flavivirus IgM antibodies was lower than for IgG, as demonstrated by less binding to the Ewt protein (Fig 6A). By using the DENV 1–4 Equad mixture the mean value of signals for DENV sera was enhanced as compared for DENV-2 Ewt. On the other hand, WNV cross-reactivity was significantly reduced in comparison to DENV-2 Ewt (Fig 6B and S2 Table).

## Discussion

A challenge for current serological dengue diagnosis is the high degree of cross-reactivity between antibodies produced during infections with related flaviviruses [27–29], leading to false positive results in currently available serological test systems [11,12]. Especially in areas with different co-circulating flaviviruses, this is of concern [1,14]. Here, we present insect-cell derived recombinant DENV E proteins bearing mutations in the conserved FL domain to enhance the specificity of serological DENV diagnosis. When using the wild type E protein from serotype 2 (which is the basis for several available test systems) we found all the DENV-positive sera displayed high signals (Fig 4A). However, the cross-reactivity problem was also confirmed, as the signals obtained with many WNV- and TBEV positive samples were in the range of DENV-signals, although the mean intensities were lower (Figs 3E and 4A). As a consequence, several sera would be misdiagnosed as false-positive, which was indeed observed when a commercial assay was used (Fig 5).

It has been shown before that a large proportion of antibodies to DENV-infections are produced against the FL domain [17,18,22]. Accordingly, two out of three dengue sera showed a clearly decreased signal when the FL-mutant version of the E-protein from DENV-2 was used (Fig 3B and 3E). However, in the larger serum panel, the signals obtained with the DENV-sera remained detectable, but most of the binding of the heterologous flavivirus-antibodies was eliminated (Figs 3B and 4B). This result confirms previous studies performed with WNV and JEV, using VLP-based systems with single- or double mutations in the FL domain [20,21] and underlines the role of the conserved FL domain in cross-reactivity between flavivirus-antibodies. It also demonstrates that the four mutations of the Equad proteins from WNV [24] can be used to eliminate cross-reactive epitopes in DENV E proteins. When the mixture of all four serotype-specific mutant proteins was used, the values of many DENV-positive serum samples increased as compared to using DENV-2 Equad only, although only half the amount of total protein was used (Fig 4C). This increase in sensitivity might result from serum samples that were from non-serotype 2 infections and now bound to their corresponding E-protein, such as the three outliers in Fig 4B. However, in the absence of knowledge of the infecting serotype, this interpretation remains speculative. Data on the infecting serotypes were available only for a limited subset of the DENV-positive sera in this study. In addition to an increase in sensitivity, the cross-reactivity of TBEV and WNV sera was further reduced with the serotype mixture and lower amount of antigen. Under the conditions used, all DENV-infected sera of the panel led to high absorbance values with the DENV 1–4 mix. Nevertheless, to avoid single outliers, the exact relative contribution and amount of each serotype mutant protein in the mixture might still be optimized. The titration curves (Fig 3) suggest that a doubling of the amount of the individual mutant antigens does still not lead to an increase in cross-reactivity.

Using the DENV 1–4 mix for IgM detection resulted in a higher mean value of positive samples as compared to the wildtype antigen (Fig 6). At the same time, the cross-reactivity with WNV-positive sera was decreased. Due to the generally observed higher specificity of IgM-antibodies [12], this improvement in specificity was less pronounced as for IgG-detection (Fig 6). Nevertheless, our data support the idea that recombinant E proteins bearing four point mutations are suitable antigens for the specific serological diagnosis of dengue. This is also supported by testing samples from a recent external quality assessment on serological dengue diagnosis [12], where the vast majority of participating laboratories (using various ELISA systems) detected a cross-reactive WNV serum as false positive. The serotype mixture used here was able to detect the samples with 100% accuracy (results are included in Fig 4), including diluted samples for sensitivity determination. In addition, the direct comparison of the DENV-1-4 mixture antigen with a commercially available test clearly showed the increased specificity of the mutant antigens (Fig 5). Although no cut-offs were yet defined for the DENV1-4 Equad, it is clear from Figs 4 and 5 that cross-reactive sera cause absorbance values more similar to flavivirus-negative than to DENV-positive samples.

We recently have performed a study using a bacterially expressed Equad mutant for WNV [24]. These data also showed a reduction in cross-reactivity. However, especially some DENV-positive samples with a high antibody titer still bound the mutant protein to some extent. Therefore, the ratio between signals obtained with the wildtype and mutant protein was calculated for each serum enabling correct diagnosis. In contrast, in the present study the mutant proteins alone were sufficient to exclude cross-reactivity, which might reflect higher sensitivity of the insect-cell derived antigen or specific differences between the antibody responses to WNV and DENV.

In summary, a system for the sensitive and specific serological diagnosis of DENV-infections is presented, which consists of recombinant E-proteins from the four major serotypes, with mutations in the FL and an adjacent loop domain. The binding of cross-reactive antibodies from heterologous flavivirus-infections is strongly decreased. These antigens might form a valuable basis for improved antibody tests, especially in areas where co-circulation of different flaviviruses is observed.

## Supporting Information

S1 Table Analysis of the recombinant DENV E proteins with monoclonal antibodies (mAb) recognizing conformational epitopes. (DOCX)

S2 Table Statistical analysis of data presented in Figs 4 and 6. (DOCX)

S3 Table Test of different antigen-amounts for measurement of IgM antibodies. (DOCX)
