# Supplementary material for: Recombinant Envelope-Proteins with Mutations in the Conserved Fusion Loop Allow Specific Serological Diagnosis of Dengue-Infections
Source: PLoS Negl Trop Dis. 2015 Nov 13;9(11):e0004218. doi: 10.1371/journal.pntd.0004218 (PMC4643925; doi:10.1371/journal.pntd.0004218)
Supplement: S1 Table — (DOCX) [file pntd.0004218.s001.docx]

**Supplementary table 1:** Analysis of the DENV E proteins with monoclonal antibodies

| **mAb** | **DENV-2 Ewt** | **DENV-2 Equad** | **DENV-1 Equad** | **DENV-3 Equad** | **DENV-4**  **Equad** |
| --- | --- | --- | --- | --- | --- |
|  |  |  |  |  |  |
| DV2-76 | 1.413 | 1.111 | - | - | - |
| DV2-96 | 1.126 | 1.052 | - | - | - |
| DV2-106 | 0.880 | 0.706 | - | - | - |
| DV2-44 | 0.980 | 0.726 | - | - | - |
| DV2-29 | 1.688 | 0.154 | - | - | - |
| WNV E18 | 2.267 | 0.149 | 0.188 | 0.300 | 0.172 |
| WNV E60 | 1.690 | 0.174 | 0.270 | 0.343 | 0.249 |

**Supplementary table 1:** analysis of the DENV E proteins with monoclonal antibodies (mAb) recognizing conformational epitopes. 2 μg/ml of protein were coated onto 96-well plates and an ELISA was performed. Values indicate absorbance (450nm) and are from one representative experiment of two.

Antibodies:

DENV-2 specific: DV2-44 (domains DI-DII); DV2-76, DV2-96, DV2-106 (domain DIII); DV2-29 (FL-domain)

WNV-specific/cross reactive: E18, E60 (FL-domain).

**References:**

Sukupolvi-Petty S, Austin SK, Engle M, Brien JD, Dowd KA, Williams KL et al. (2010) Structure and function analysis of therapeutic monoclonal antibodies against dengue virus type 2. Journal of virology. 84(18):9227-9239. doi: 10.1128/JVI.01087

Oliphant T, Nybakken GE, Engle M, Xu Q, Nelson CA, Sukupolvi-Petty S et al. (2006) Antibody recognition and neutralization determinants on domains I and II of West Nile Virus envelope protein. Journal of virology 80(24):12149-12159.
