# Supplementary material for: Recombinant Envelope-Proteins with Mutations in the Conserved Fusion Loop Allow Specific Serological Diagnosis of Dengue-Infections
Source: PLoS Negl Trop Dis. 2015 Nov 13;9(11):e0004218. doi: 10.1371/journal.pntd.0004218 (PMC4643925; doi:10.1371/journal.pntd.0004218)
Supplement: S2 Table — (DOCX) [file pntd.0004218.s002.docx]

**Supplementary table 2: statistical analysis**

**Figure 4A**

| *sera* | *mean* | *STDV* |
| --- | --- | --- |
| DENV | 2.364 | 0.480 |
| WNV | 1.261 | 0.594 |
| TBEV | 0.821 | 0.588 |
| YFV | 0.149 | 0.081 |
| NEG | 0.218 | 0.217 |

**Figure 4B**

| *sera* | *mean* | *STDV* | *difference to*  *DENV-2 Ewt ^§^* |
| --- | --- | --- | --- |
| DENV | 1.896 | 0.702 | ** |
| WNV | 0.106 | 0.059 | ** |
| TBEV | 0.160 | 0.138 | ** |
| YFV | 0.033 | 0.01 | ** |
| NEG | 0.075 | 0.033 | NS |

**Figure 4C**

| *sera* | *mean* | *STDV* | *difference to*  *DENV-2 Ewt ^§^* | *difference to*  *DENV-2 Equad ^§^* |
| --- | --- | --- | --- | --- |
| DENV | 1.816 | 0.549 | ** | NS |
| WNV | 0.063 | 0.031 | ** | * |
| TBEV | 0.084 | 0.058 | ** | NS |
| YFV | 0.047 | 0.018 | * | NS |
| NEG | 0.052 | 0.023 | * | NS |

**Figure 5A**

| *sera* | *mean* | *STDV* |
| --- | --- | --- |
| DENV | 0.852 | 0.388 |
| WNV | 0.265 | 0.189 |
| NEG | 0.072 | 0.095 |

**Figure 5B**

| *sera* | *mean* | *STDV* | *difference to*  *DENV-2 Ewt ^§^* |
| --- | --- | --- | --- |
| DENV | 1.249 | 0.691 | * |
| WNV | 0.112 | 0.059 | * |
| NEG | 0.067 | 0.062 | NS |

§: Mann-Whitney Rank Sum Test. The Two asterisks (**) indicate statistical significance with

P < 0,001; one asterisk (*) indicates P < 0,05. NS: not significant.
