# Supplementary material for: Recombinant Envelope-Proteins with Mutations in the Conserved Fusion Loop Allow Specific Serological Diagnosis of Dengue-Infections
Source: PLoS Negl Trop Dis. 2015 Nov 13;9(11):e0004218. doi: 10.1371/journal.pntd.0004218 (PMC4643925; doi:10.1371/journal.pntd.0004218)
Supplement: S3 Table — (DOCX) [file pntd.0004218.s003.docx]

**Supplementary table 3**: test of different antigen-amounts for measurement of IgM antibodies

**A**

| **DENV-2 Equad** | **200 ng** | **300ng** | **400ng** |
| --- | --- | --- | --- |
| Serum 1 (pos) | 0.354 | 0.395 | 0.465 |
| Serum 2 (pos) | 1.114 | 1.447 | 1.512 |
| Serum 3 (neg) | 0.139 | 0.152 | 0.181 |

**B**

| **DENV 1-4 Equad Mix** | **100 ng** | **200 ng** | **300 ng** |
| --- | --- | --- | --- |
| Serum 1 (pos) | 0.172 | 0.351 | 0.549 |
| Serum 2 (pos) | 0.158 | 0.421 | 0.982 |
| Serum 3 (neg) | 0.121 | 0.124 | 0.127 |

**Supplementary table 2**: test of different antigen-amounts for measurement of IgM antibodies against DENV. Indicated amounts of antigens per well were coated in 96-well plates and an ELISA was performed according to the Materials and Methods section. A, DENV-2 Equad protein; B, DENV1-4 Equad mix. Values indicate absorbance (450nm) and are from one representative experiment of two. Sera were from DENV-infected (pos) or not infected (neg) individuals.
